# Involvement of Virus-Induced Interferon Production in IgG Autoantibody-Mediated Anemia

**DOI:** 10.3390/ijms22169027

**Published:** 2021-08-21

**Authors:** Sarah Legrain, Dan Su, Mélanie Gaignage, Cor Breukel, Jill Claassens, Conny Brouwers, Margot M. Linssen, Shozo Izui, J. Sjef Verbeek, Jean-Paul Coutelier

**Affiliations:** 1 Unit of Experimental Medicine, de Duve Institute, Université Catholique de Louvain, 1200 Bruxelles, Belgium; sarah.legrain.phd@gmail.com (S.L.); sudan20072007@yahoo.com (D.S.); melanie.gaignage@uclouvain.be (M.G.); 2 Department of Human Genetics, Leiden University Medical Center, 2300 RC Leiden, The Netherlands; C.Breukel@lumc.nl (C.B.); J.W.C.Claassens@lumc.nl (J.C.); C.M.Brouwers@lumc.nl (C.B.); M.M.L.Linssen@lumc.nl (M.M.L.); j.s.verbeek@toin.ac.jp (J.S.V.); 3 Department of Pathology and Immunology, Centre Médical Universitaire, University of Geneva, 4 1211 Geneva, Switzerland; shozo.izui@gmail.com

**Keywords:** Fc receptors, lactate dehydrogenase-elevating virus, autoimmune anemia, interferon

## Abstract

Infection with viruses, such as the lactate dehydrogenase-elevating virus (LDV), is known to trigger the onset of autoimmune anemia through the enhancement of the phagocytosis of autoantibody-opsonized erythrocytes by activated macrophages. Type I interferon receptor-deficient mice show enhanced anemia, which suggests a protective effect of these cytokines, partly through the control of type II interferon production. The development of anemia requires the expression of Fcγ receptors (FcγR) I, III, and IV. Whereas LDV infection decreases FcγR III expression, it enhances FcγR I and IV expression in wild-type animals. The LDV-associated increase in the expression of FcγR I and IV is largely reduced in type I interferon receptor-deficient mice, through both type II interferon-dependent and -independent mechanisms. Thus, the regulation of the expression of FcγR I and IV, but not III, by interferons may partly explain the exacerbating effect of LDV infection on anemia that results from the enhanced phagocytosis of IgG autoantibody-opsonized erythrocytes.

## 1. Introduction

Mouse infection with the lactate dehydrogenase-elevating virus (LDV) has been shown to exacerbate autoimmune diseases, such as antibody-mediated anemia [1,2] and thrombocytopenia [3,4]. This effect of LDV infection can be explained by the enhanced phagocytic activity of macrophages [1,3], thus leading to the engulfment and destruction of opsonized cells in response to the macrophage activation, which is mostly triggered by the gamma-interferon (IFN-γ) secreted by natural killer cells upon infection [5]. Thus, LDV provides a useful experimental model to study the role of viral infections and, more precisely, the impact of their triggered cytokine secretion in the pathogenesis of autoantibody-mediated blood autoimmune diseases, such as immune thrombocytopenic purpura and hemolytic anemia.

The respective roles of Fc receptors (FcRs) and antibody isotypes in the phagocytosis of opsonized erythrocytes have been extensively analyzed in uninfected animals through the use of a large panel of anti-red blood cell antibody switch variants and mice deficient in one or several FcRs for IgG (FcγR) [6,7,8,9]. These studies indicate that all anti-erythrocyte antibody isotypes can be pathogenic at different levels, depending on their affinity and dose as well as on their interactions with the various activating FcRs and with complement. Interestingly, all activating FcγRs (FcγR I, FcγR III, and FcγR IV) can mediate the phagocytosis of erythrocytes opsonized by IgG2a, a major autoantibody isotype produced in mouse experimental models [10,11,12], leading to anemia [9]. The increased capacity of macrophages from LDV-infected mice to bind and ingest opsonized targets was explained by a virus-induced enhancement of FcγR expression on these cells [13], but this has never been directly demonstrated. Thus, the present study was performed to analyze the effects of LDV infection on the expression of all activating FcγRs, their involvement in the exacerbated phagocytic activity, and the resulting anemia triggered by infection.

## 2. Results

### 2.1. Role of Type I and Type II IFNs in Red Blood Cell (RBC) Phagocytosis in LDV-Infected Mice

**Figure 1:**
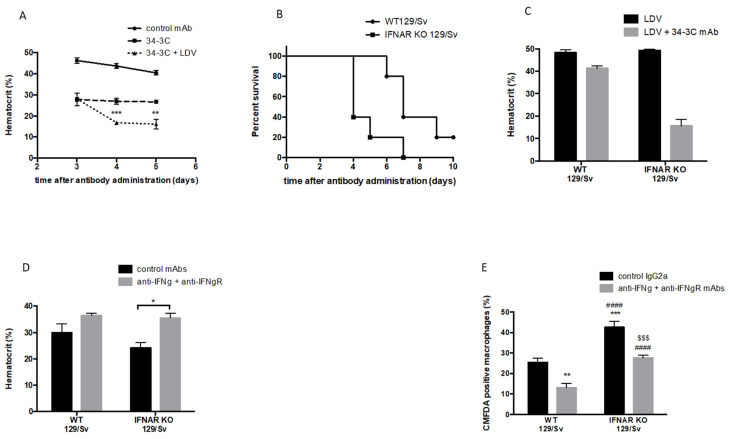
Role of type I and type II IFNs in anemia and ex vivo erythrophagocytosis in LDV-infected mice. (**A**) Hematocrits in groups of 6 to 8 C57BL/6 mice pooled from 2 independent experiments at different times after administration of 50 μg 34-3C IgG2a mAb or A6202F4M control mAb. LDV infection occurred one day before mAb administration. (**B**) Survival of 10 129/Sv or IFNAR KO-infected mice after administration of 350 μg 34-3C anti-RBC mAb. (**C**) Hematocrit in groups of WT 129/Sv or IFNAR KO mice challenged with 50 μg of 34-3C IgG2a mAb or A6202F4M control mAb. Hematocrit were measured 4 days after LDV infection. (**D**) Hematocrits after administration of 50 μg 34-3C IgG2a mAb to groups of 4 129/Sv or IFNAR KO-infected mice treated with or without IFN-γ and IFN-γR-blocking antibodies (300 μg one day before LDV infection, 1 mg 3 h after infection, followed by a dose of 1 mg every 2 days). (**E**) Ex vivo erythrophagocytosis of 34-3C-opsonized RBCs by macrophages of 9 to 11 129/Sv or IFNAR KO-infected mice pooled from 2 independent experiments and treated with or without IFN-γ and IFN-γR-blocking antibodies (300 μg one day before LDV infection, 500 μg 3 h after infection). * *p* < 0.05, ** *p* < 0.01, *** *p* < 0.001, * compared to infected 129/Sv; $$$ compared to anti-IFNγ-treated infected 129/Sv; #### compared to IFNAR KO. The results are shown as the means ± SEM.

To demonstrate the effect of viral infection on the establishment of autoimmune hemolytic anemia, 34-3C anti-RBC IgG2a mAb was injected into LDV-infected mice. The administration of 50 μg of this antibody to wild-type (WT) C57BL/6 mice induced moderate anemia after 4 and 5 days compared to WT animals treated with the control IgG2a (Figure 1A), and LDV infection worsened the anemia developed by 34-3C-treated mice (Figure 1A, *p* = 0.0005 and *p* < 0.0081, respectively). Interestingly, type I IFNs, which are produced early on in the course of infection [14], seem to protect mice from severe anemia, as LDV-infected IFNAR KO 129/Sv mice that received a large amount of 34-3C mAb (350 µg) showed decreased survival compared to the control WT 129/Sv mice (Figure 1B, *p* = 0.009). This correlated with higher anemia in type I IFN-deficient animals 4 days after the antibody infusion (Figure 1C, *p* = 0.0286, compared to the WT 129/Sv counterparts) and was prevented with a blockade of IFN-γ signaling in IFNAR KO mice (Figure 1D, *p* = 0.0286). The ex vivo erythrophagocytosis of CMFDA-labeled and 34-3C opsonized RBCs by peritoneal macrophages isolated from infected IFNAR KO mice was increased compared to macrophages from WT 129/Sv mice (Figure 1E, *p* = 0.0002). In contrast, a blockade of IFN-γ signaling reduced ex vivo erythrophagocytosis by WT and IFNAR KO macrophages (Figure 1E, *p* = 0.0015 and *p* = 0.0003, respectively), which corroborates the in vivo data. Altogether, these results suggest a protective role of type I IFNs in the development of LDV-exacerbated autoimmune anemia thwarted by type II IFN.

### 2.2. Involvement of FcγRs in In Vivo and Ex Vivo LDV-Exacerbated Erythrophagocytosis

**Figure 2:**
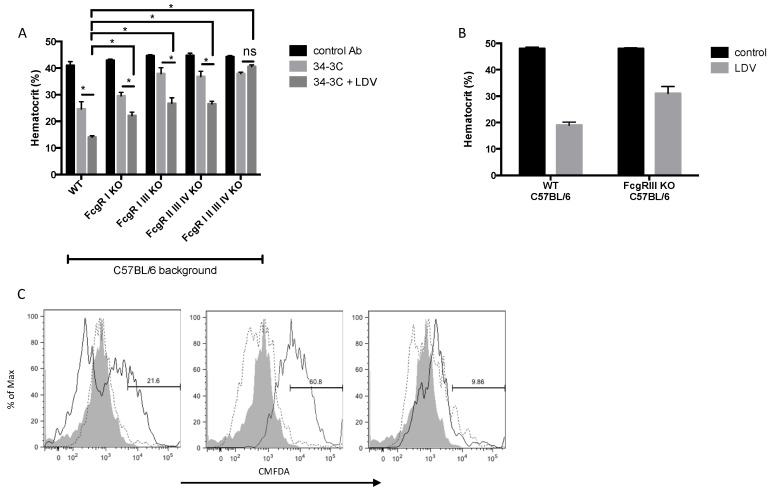
Involvement of Fcγ receptors in LDV-exacerbated in vivo and ex vivo erythrophagocytosis. (**A**,**B**) Hematocrits of C57BL/6, FcγR I KO, FcγR I/III KO, FcγR II/III/IV KO, FcγR I/II/III/IV KO (**A**), and FcγR III KO (**B**) mice, 4 days after administration of 50 µg of 34-3C mAb. LDV infection was performed one day before the antibody challenge. The results are representative of at least 2 experiments and shown as the means ± SEM, ). * *p* < 0.05. (**C**) Ex vivo erythrophagocytosis of non-opsonized (dashed line) or 34-3C-opsonized (plain line) CMFDA-stained RBCs by macrophages isolated from (left panel) mock-infected C57BL/6, (middle panel) LDV-infected C57BL/6, and (right panel) LDV-infected FcγR I/II/III/IV KO (grey: fluorescence of macrophages without RBC incubation). The results are representative of at least 2 independent experiments.

The hematocrits of LDV-infected WT mice and mice deficient in FcγRs were compared 4 days after the administration of a moderate dose of 34-3C mAb (Figure 2A,B). In uninfected animals, the absence of FcγR III (FcγR I/III, FcγR II/III/IV, and FcγR I/II/III/IV KO mice) led to strongly reduced anemia compared to the WT animals (Figure 2A). In addition, a minor role of FcγR I was suggested by a limited reduction in the disease in animals deficient in this receptor. Finally, LDV-infected FcγR KO mice were completely resistant to 34-3C-induced hemolytic anemia (Figure 2A, *p* < 0.0001), whereas the depletion of the complement protein C3 through the administration of cobra venom factor (CVF) did not decrease the development of anemia in LDV-infected 34-3C-treated WT mice (Figure S1). These results suggest a C3-independent but FcγR-dependent mechanism of RBC phagocytosis on LDV infection. The anemia developed in LDV-infected FcγR I, FcγR I/III, and FcγR II/III/IV KO mice was partially inhibited compared to the anemia in WT animals, but not as strongly as in FcγR I/II/III/IV mice (Figure 2A). In addition, the anemia of 34-3C-treated FcγR III KO mice increased after LDV infection but did not reach the same severity as the anemia of their WT counterparts (Figure 2B, *p* = 0.0364). Together, these results point to the involvement of all activating FcγRs in virally exacerbated anemia induced by the 34-3C autoantibody.

The ex vivo erythrophagocytosis of CMFDA-stained 34-3C-opsonized RBCs by peritoneal macrophages harvested from LDV-infected C57BL/6 mice increased compared to their counterparts obtained from mock-infected controls (Figure 2C, middle vs. left panel, *p* = 0.0047), and this enhanced erythrophagocytic activity was abolished in macrophages harvested from infected FcγRI/II/III/IV quadruple KO mice (Figure 2C, middle vs. right panel, *p* = 0.0006). These in vitro results are in agreement with our in vivo data, demonstrating that in the absence of all FcγRs, erythrophagocytosis is abolished.

### 2.3. Modification of Fcγ Receptor Expression by LDV Infection

**Figure 3:**
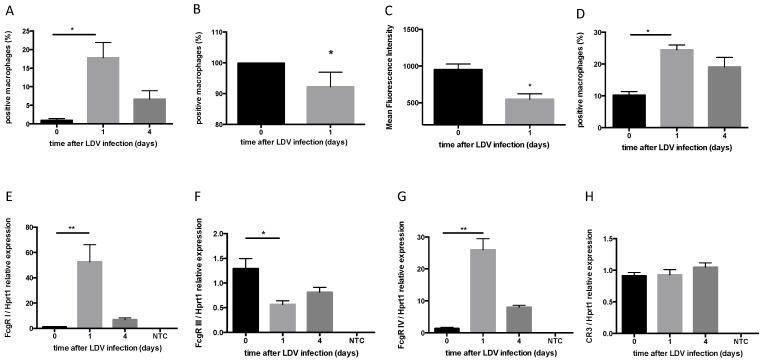
Modulation of activating Fcγ receptors and of CR3 expression after LDV infection. (**A**–**D**) The CD11b+ F4/80+ peritoneal macrophages of 4 mock- or LDV-infected C57BL/6 were stained with either anti-FcγR I (**A**), anti-FcγR III (**B**,**C**), or anti-FcγR IV (**D**) antibodies. (**E–H**) Fcγ (**E**–**G**) and C3 (**H**) receptor gene expression in CD11b+ F4/80+ peritoneal macrophages from groups of 4 mock- or LDV-infected C57BL/6 were assessed by RT-qPCR. The results are shown as the means ± SEM. NTC: negative control. * *p* < 0.05, ** *p* < 0.01.

The expression of activating FcγRs was measured through flow cytometry on CD11b^+^ F4/80^+^ peritoneal macrophages. LDV infection induced an increase in the population of macrophages expressing FcγR I and FcγR IV one day after infection (Figure 3A, *p* = 0.0286 and Figure S2A; Figure 3D, *p* = 0.0286, and Figure S2C, respectively). This correlated with a strong increase in the mRNA expression of both receptors (Figure 3E, *p* = 0.0062 and Figure 3G, *p* = 0.0092, respectively) one day post-infection. In contrast, LDV slightly decreased both the number of peritoneal macrophages expressing FcγR III and the intensity of FcγR III expression in these cells (Figure 3B, *p* = 0.0286 and Figure 3C, *p* = 0.0286, respectively, and Figure S2B). This decrease in FcγR III membrane protein expression correlated with a decreased expression of FcγR III mRNA (Figure 3F, *p* = 0.0038). In contrast, the expression of CR3 mRNA did not change in the LDV-infected mice compared to the mock-infected animals (Figure 3H).

### 2.4. Effect of Type I and Type II IFNs on FcγR Expression

**Figure 4:**
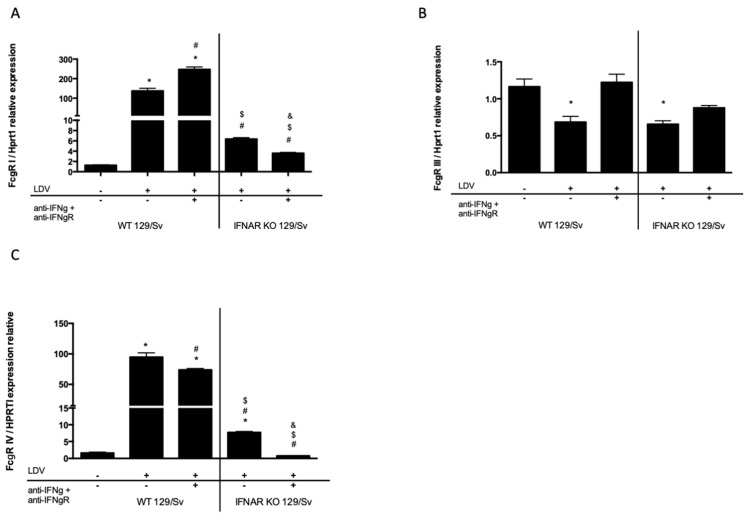
Role of type I and type II IFNs in Fcγ receptor expression in peritoneal macrophages of LDV-infected mice. The expressions of FcγR I (**A**), FcγR III (**B**), and FcγR IV (**C**) were measured by RT-qPCR in macrophages isolated from groups of 4 129/Sv or IFNAR KO mice 1 day after LDV infection. * compared to control 129/Sv; # compared to infected 129/Sv; $ compared to infected 129/Sv treated with IFN-γ and IFN-γR-blocking antibodies. & compared to infected IFNAR KO treated with IFN-γ and IFN-γR-blocking antibodies. The results are shown as the means ± SEM.

IFNAR KO mice were used to determine the role of type I IFNs in the modulation of FcγR expression after LDV infection. The expression of FcγR I mRNA strongly decreased in LDV-infected IFNAR KO mice compared to the expression of FcγR I mRNA in the WT counterparts (Figure 4A, *p* = 0.0286). Treatment with a cocktail of anti-IFN-γ and anti-IFN-γR mAbs resulted in a moderate increase in the expression of FcγR I in the WT (Figure 4A, *p* = 0.086) but not the IFNAR KO mice. The expression of FcγR IV mRNA was also reduced in infected IFNAR KO mice compared to their WT counterparts (Figure 4C). However, in contrast to its enhancing effect on FcγR I mRNA expression, the IFN-γ blockade slightly decreased FcγR IV mRNA expression in both WT and IFNAR KO mice (Figure 4C). Decreased FcγR III mRNA expression was prevented with the blockade of IFN-γ signaling (Figure 4B, *p* = 0.0286).

## 3. Discussion

Strong evidence has been reported that LDV exacerbates diseases, such as thrombocytopenia and hemolytic anemia by increasing the uptake of target cells by macrophages [1,2,3,4]. Although platelets or erythrocytes are most often ingested after opsonization with IgG autoantibodies, LDV infection alone may also result in antibody-independent thrombocytopenia [15]. Other mouse viruses, including the mouse hepatitis virus (MHV) [3] and the lymphocytic choriomeningitis virus [16], have also been reported to induce macrophage activation, leading to the enhanced destruction of opsonized platelets or erythrocytes. Such a pathogenic mechanism may thus contribute to human autoimmune diseases following infection with common viruses, such as immune thrombocytopenic purpura and hemolytic anemia, which have also been recently reported after infection with SARS-CoV-2 [17,18,19].

Viral infections are known to trigger the secretion of several IFN types, including IFN-γ, which is secreted by NK cells after LDV and MHV infection [5,20] and which mediates most observed macrophage activation. Thus, IFN-γ plays a major role in the exacerbation of antibody-mediated thrombocytopenia and anemia induced by viruses, such as LDV [2,3,4,21], as well as in enhanced sensibility to endotoxin shock [22]. In contrast, we found that type I IFNs protect against autoantibody-mediated macrophage uptake and the resulting anemia. A similar protective effect against LDV-induced sensitization to endotoxin shock was also reported [22]. Since type I IFNs modulate LDV-induced IFN-γ secretion [23], it may be postulated that their protective effect is mostly indirect. However, an additional direct regulating effect on macrophages could not be excluded.

As expected, the pathogenicity of an IgG2a anti-erythrocyte autoantibody, the most frequent isotype found in experimental models of anemia, depends on Fcγ receptor expression in macrophages. It was reported that the induction of severe anemia by a high dose of an IgG2a anti-erythrocyte autoantibody is dependent on FcγR I and FcγR IV, whereas the induction of mild anemia by a low dose of the same antibody is dependent on FcgR III [9]. Using a panel of FcγR KO mice, we found that the absence of FcγR I led to reduced anemia induced by IgG autoantibodies in LDV-infected animals, and that merely the expression of FcγR I allowed for the development of anemia, although it was less severe compared to the disease induced in WT mice receiving the same autoantibody. Moreover, severe anemia triggered by autoantibody administration to LDV-infected mice appeared to also depend on the concomitant expression of both FcγR III and FcγR IV. Therefore, it could be postulated that the increased uptake of opsonized erythrocytes by macrophages from LDV-infected mice might correlate with an increased expression of activating FcγRs. This was the case for FcγR I and FcγR IV. In contrast, macrophage FcγR III expression decreased after LDV infection, although its functional involvement can be demonstrated. This observation, confirming the role of FcγR III in autoantibody-induced anemia [24], can possibly be explained by an increased affinity of this receptor for IgG2a Ab; however, this would need to be confirmed through ligand-binding assays. Such a higher affinity of the IgG2a isotype has been reported for FcγR III of C57BL/6 mice, compared with IgG2c—the equivalent isotype of these animals—although both IgG2a and IgG2c strongly reacted with FcγR I, FcγR III and FcγR IV [25]. Another possible explanation is that FcγR expression in peritoneal macrophages differs from that in Kupfer cells, the main cell type involved in the phagocytosis of IgG-opsonized erythrocytes in the liver [26]. Thus, the severe anemia triggered by autoantibody administration to LDV-infected mice appeared to depend on the concomitant expression of all three activating receptors: FcγR I, FcγR III, and FcγR IV. FcγR I expression was previously shown to be induced by IFN-γ [27]. However, the strong increase in FcγR I expression observed in WT mice after LDV infection was not suppressed by treatment with neutralizing anti-IFN-γ mAb, whereas enhanced FcγR IV expression only moderately decreased with this treatment. This might be due to an activating effect of other cytokines produced in response to LDV infection, or to an insufficient neutralizing effect of the mAb due to the large amounts of IFN-γ resulting from this infection. In addition, type I IFNs appear to also contribute to the enhanced expression of both FcγR I and FcγR IV expression after LDV infection, since this expression was largely reduced in type I IFN receptor KO mice. Moreover, in these animals, the neutralization of IFN-γ further reduced the enhancement in FcγR I expression by LDV infection and completely abolished the enhancement in FcγR IV expression by LDV infection, suggesting that type I and II IFNs are concomitantly involved in this effect of the virus.

Therefore, the enhancement in FcγR I and FcγR IV expression resulting from type I and II IFN secretion might play an important role in the severe anemia induced in infected animals. However, since type I IFN appears to induce opposite effects on FcγR expression and RBC destruction, mechanisms other than FcγR modulation might also play a role in the exacerbating effect of the virus on autoantibody-mediated anemia.

## 4. Materials and Methods

### 4.1. Mice and Virus

Specific pathogen-free C57BL/6, IFN-α/ßR^-/-^ (IFNAR KO) (PMID 8009221), and WT 129/Sv mice were bred at the Ludwig Institute for Cancer Research (Brussels, Belgium) by G. Warnier. All the mice used were aged between 8 and 12 weeks old. The project was approved by the local commission for animal care.

The mice were infected with an intraperitoneal injection of approximately 2 × 10^7^ 50% infectious doses (ID_50_) of LDV (Riley strain; ATCC, Manassas, VA) in 500 µL saline.

FcγR I KO (Fcgr1^tm1Jsv^, MGI 2664927), FcγR III KO (Fcgr3^tm1Jsv^, MGI:1861924), FcγR II/III/IV KO [28], and FcγR I/II/III/IV KO mice [29] on the C57BL/6 background were kindly provided by the laboratory of Sjef Verbeek in the Netherlands. The mice were mated at the De Duve Institute by S. Legrain, and the pups were genotyped using the appropriate primers.

### 4.2. Antibodies and Drugs

The CRL-2024 anti-IFN-γ receptor, F3 anti-IFN-γ, 34-3C anti-red blood cells (RBCs), IgG2a mAbs, and 2.4G2 anti-FcγR IIb/III IgG2b mAb (gamma block) were purified on protein G sepharose beads from hybridoma cultures. PE anti-mouse CD64 mAb (FcγR I), PE isotype control, PE anti-mouse F4/80, APC anti-mouse F4/80, PerCP anti-mouse CD11b mAbs, and Alexa 647 anti-mouse FcγR IV mAbs were purchased from Biolegend (San Diego, CA, USA). PE anti-mouse CD16 (FcγR III) was purchased from R&D Systems (Minneapolis, MN, USA). Normal hamster IgG (eBioscience, San Diego, CA, USA) was used as a control isotype. Cobra Venom Factor (CVF; Quidel Corporation, San Diego, CA, USA) was used at a rate of 4 U/mouse.

### 4.3. Isolation and Culture of Peritoneal Cells

Resident peritoneal macrophages were harvested by washing the peritoneal cavity with ice-cold PBS supplemented with 5% fetal bovine serum (FBS, Gibco, Life Technologies, Grand Isle, NY, USA), 50 U/mL penicillin G, 50 μg/mL streptomycin (Gibco, Life Technologies), and 2 mM EDTA (Sigma Aldrich, St. Louis, MO, USA). The cells were washed, resuspended in Iscove’s Modified Dulbecco’s Medium (IMDM, Gibco Life Technologies) supplemented with 10% FBS, non-essential amino acids, 50 U/mL penicillin G, and 50 μg/mL streptomycin, and plated in a 6-well tissue culture plate (Corning Inc., Corning, NY, USA). After 2 h of incubation at 37 °C and 7% CO_2_, the non-adherent cells were washed away and the adherent peritoneal macrophages were processed.

### 4.4. Type I and Type II IFN Stimulation

Adherent peritoneal macrophages were cultured in IMDM, supplemented as described above, containing 100 U/mL of either IFN-α, IFN-β (Biolegend, San Diego, CA, USA), or both for 24 h. When indicated, 20 U/mL of IFN-γ (Biolegend, San Diego, CA, USA) was added for 48 h following the type I IFN stimulation.

### 4.5. Hematocrit

Blood was collected from the retro-orbital plexus of the anesthetized mice, and the hematocrits were measured after the centrifugation of heparinized blood in a Hettich Haematokrit centrifuge (Tuttlingen, Germany).

### 4.6. Flow Cytometry

Flow cytometry analysis was performed as follows: peritoneal adhering cells were incubated with 2.4G2 (gamma block) mAb prior to incubation with either PE anti-mouse FcγR I, PE anti-mouse FcγR III, or Alexa 647 anti-mouse FcγR IV antibodies. PerCP anti-mouse CD11b and APC or PE anti-mouse F4/80 staining were performed simultaneously to label the macrophages. Fluorescence was acquired with an LSR Fortessa flow cytometer (Becton Dickinson, Franklin Lakes, NJ, USA) and analyzed using FlowJo Software 9.8.1 (Tree Star Inc., Ashland, OR, USA).

### 4.7. Gene Expression Analysis

The total RNA was isolated from peritoneal adhering cells using a Reliaprep RNA Cell Miniprep System from the Promega Corporation (Fitchburg, WI, USA). RNA purity was measured using a Nanodrop 6000, and RNA integrity was assessed with 1% agarose gel electrophoresis. Then, 1 μg of total RNA was reverse-transcribed using RevertAid H minus Reverse Transcriptase (Thermo Scientific, Waltham, MA, USA) and oligo dT as a primer, according to the manufacturer’s instructions.

Gene expression was measured by RT-qPCR with an Eco Real-Time thermocycler (Illumina, San Diego, CA, USA), using a Core Kit for SYBR Green I (Eurogentec, Liege, Belgium). The primers for FcγR I [21], FcγR III, FcγR IV [22], and CR3 [23] are described elsewhere. HPRT1 gene expression was used as a reference gene (forward: 5′-TAATCACGACGCTGGGACTG-3′, and reverse: 5′-GTTGGGCTTACCTCACTGCT-3′). The HPRT1 primers were designed using a Primer-BLAST (NCBI, Bethesda, MD, USA) and were validated for stability among 6 references genes using GeNorm software (Biogazelle, Zwijnaarde, Belgium). The ratios between the genes of interest and HPRT1 expression were calculated using the Eco Real-Time qPCR software (Illumina, San Diego, CA, USA).

### 4.8. Ex Vivo Erythrophagocytosis

A total of 40 × 10^6^ control or 34-3C-opsonized CMDFA-labeled RBCs were incubated for 2 h at 37 °C with 3 × 10^6^ adherent peritoneal macrophages. After 2 h, non-phagocytosed RBCs were washed away twice with 1 mL of RT 1× PBS. The adherent peritoneal macrophages were lifted up in 1 mL of ice-cold 1× PBS-BSA 0.5% supplemented with 2 mM EDTA + 1% FBS and gentle cell scraping. Sticking RBCs were lysed by osmotic shock (ACK lysis buffer) before resuspension in HCF + 1% FBS. Dying cells were then stained with TO-PRO^®^-3 (Life Technologies, Grand Isle, NY, USA) before acquisition on an LSR Fortessa flow cytometer (Becton Dickinson, Franklin Lakes, NJ, USA).

### 4.9. Statistical Analysis

The results are expressed as the means ± the standard error of the mean (SEM). When appropriate, Mann–Whitney and Kruskal–Wallis tests were performed using Prism 6 (La Jolla, CA, USA).

## Data Availability

Data available on request.

## Supplementary Materials

The following are available online at https://www.mdpi.com/article/10.3390/ijms22169027/s1.
