# Supplementary material for: Involvement of Virus-Induced Interferon Production in IgG Autoantibody-Mediated Anemia
Source: Int J Mol Sci. 2021 Aug 21;22(16):9027. doi: 10.3390/ijms22169027 (PMC8396558; doi:10.3390/ijms22169027)
Supplement: Supplementary file 1 [file ijms-22-09027-s001.zip › ijms-1299186-SI.pdf]

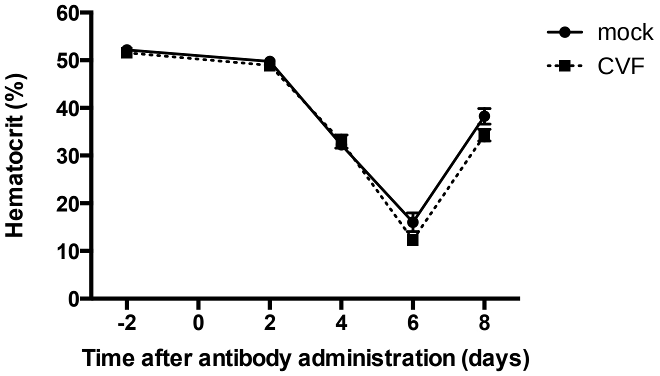

Figure S1. Effect of C3 signaling blockade on autoimmune anemia in WT mice C57BL/6 mice were infected 1 day prior to the administration of 50 µg of 34-3C mAb intraperitoneally and were given 4 U of Cobra venom factor (CFV) in PBS 2 days prior to antibody challenge and every other day throughout the experiment. Hematocrite was measured at several timepoints. Results showed are representative of 2 independent experiments.

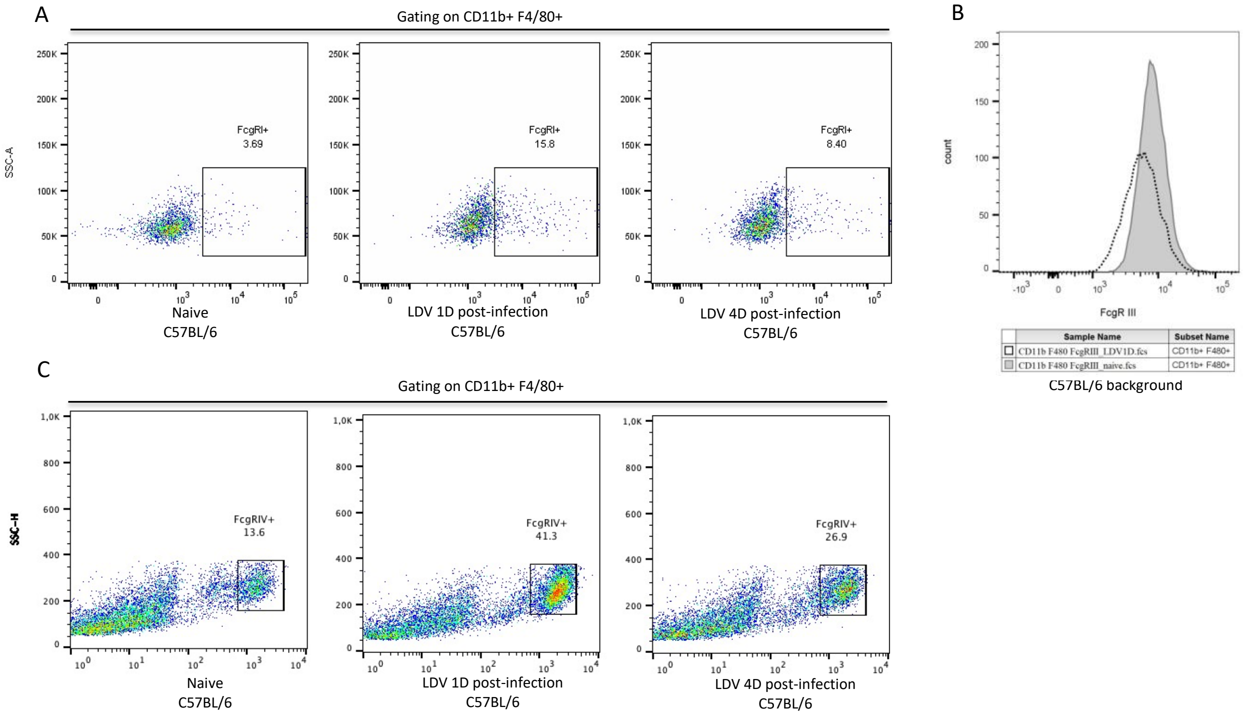

Figure S2. Modulation of activating Fcγ receptors after LDV infection. A, C. Flow cytometry dot plot gating on CD11b+ F4/80+ peritoneal macrophages of mock (left panel) or LDV-infected mice 1 day (middle panel) and 4 days (right panel) post-infection. B. Histogram of flow cytometry staining gating on CD11b+ F4/80+ peritoneal macrophages from naive (plain line) and LDV-infected mice 1day post infection (dotted line).
